# Lymphoma risk in systemic lupus: effects of treatment versus disease activity

**DOI:** 10.1186/ar3950

**Published:** 2012-09-27

**Authors:** AE Clarke, S Bernatsky, KH Costenbader, MB Urowitz, DD Gladman, PR Fortin, M Petri, S Manzi, DA Isenberg, A Rahman, D Wallace, C Gordon, C Peschken, MA Dooley, EM Ginzler, C Aranow, SM Edworthy, O Nived, S Jacobsen, G Ruiz-Irastorza, E Yelin, SG Barr, L Criswell, G Sturfelt, L Dreyer, I Blanco, L Gottesman, CH Feldman, R Ramsey-Goldman

**Affiliations:** 1 Research Institute of the McGill University Health Centre, Montreal, QC, Canada; 2 Brigham and Women's Hospital, Harvard Medical School, Boston, MA, USA; 3 Toronto Western Hospital and University of Toronto, Toronto, ON, Canada; 4 University of Laval, QC, Canada; 5 Johns Hopkins University School of Medicine, Baltimore, MD, USA; 6 West Penn Allegheny Health System, Pittsburgh, PA, USA; 7 University College of London, UK; 8 Cedars-Sinai/UCLA, Los Angeles, CA, USA; 9 University of Birmingham, UK; 10 University of Manitoba, Winnipeg, MB, Canada; 11 University of North Carolina at Chapel Hill, NC, USA; 12 SUNY - Downstate Medical Center, Brooklyn, NY, USA; 13 Feinstein Institute for Medical Research, Manhasset, NY, USA; 14 The University of Calgary, AB, Canada; 15 University Hospital - Lund, Sweden; 16 Copenhagen University Hospital, Copenhagen, Denmark; 17 Hospital de Cruces, UPV/EHU, Barakaldo, Spain; 18 University of California San Francisco, CA, USA; 19 Albert Einstein College of Medicine, Bronx, NY, USA; 20 Northwestern University Feinberg School of Medicine, Chicago, IL, USA

## Background

We recently evaluated the risk of malignancy in SLE by linking a multi-site international SLE cohort with regional tumor registries. Across 28 centers, 15,980 patients were observed for 119,846 (average 7.5) person-years. In total, 641 cancers occurred, for an overall standardized incidence ratio (SIR) of 1.14 (95% CI = 1.06 to 1.24). Hematologic malignancies were substantially increased (SIR = 3.01, 95% CI = 2.47 to 3.62), particularly non-Hodgkin's lymphoma (NHL; SIR = 4.36, 95% CI = 3.43 to 5.47) and leukemia (SIR = 1.76, 95% CI = 1.04 to 2.78) [1]. Yet the relative influence of treatment versus disease activity is unknown. Our objective was to determine the relative importance of drugs versus disease activity in mediating the increased risk of lymphoma.

## Methods

We performed case-cohort analyses within this multi-site SLE cohort. Adjusted hazard ratios (HRs) for lymphoma were generated in multivariate regression models, for time-dependent exposures to immunomodulators (cyclophosphamide, azathioprine, methotrexate, mycophenolate, antimalarials, glucocorticoids), disease activity (mean adjusted SLEDAI-2K), demographics, calendar year, Sjögren's syndrome, and SLE duration. Partially adjusted models were also performed, using only covariates whose HR CI excluded the null. Sensitivity analyses were performed, lagging cyclophosphamide exposures by 5 years. Medications were treated both categorically (ever/never) and as cumulative doses.

## Results

We studied 64 lymphomas (61 NHL, three Hodgkin's) and 4,739 cancer-free controls. As in the general population, lymphoma risk in SLE was higher in males and with age. Lymphomas occurred a mean of 13.1 years (standard deviation 9.8) after SLE diagnosis. Univariate analyses suggested a decreased lymphoma risk within the highest tertile of disease activity (relative to those with the lowest activity) but in fully adjusted models (using all variables listed above), the CI included the null (Table 1). Sensitivity analyses, lagging cyclophosphamide exposures, yielded similar results. In a partially adjusted model (retaining age and highest tertile of disease activity), the HR suggested a twofold lymphoma risk after cyclophosphamide. Despite a trend towards greater cyclophosphamide use in cases versus controls, in fully adjusted models, no drug was estimated to be an independent risk factor. Still, due to correlation, it remains difficult to differentiate the effects of medications from disease activity.

**Table 1:** Results of univariate and multivariate models assessing HR of exposures on lymphoma development in SLE patients

Variable	Univariate HR (95% CI)	Partially adjusted model (95% CI)	Multivarate HR (95% CI)
Cyclophosphamide (CY) ever	1.73 (0.90 to 3.33)	1.99 (1.00 to 3.96)	1.95 (0.61 to 6.22)
Cumulative CY >6 g	1.51 (0.63 to 3.62)	-	0.79 (0.18 to 3.54)
Azathioprine (AZA) ever	0.82 (0.45 to 1.52)	-	1.28 (0.53 to 3.07)
Cumulative AZA >36.5 g	0.49 (0.19 to 1.25)	-	0.46 (0.14 to 1.54)
Methotrexate ever used	0.99 (0.45 to 2.16)	-	0.79 (0.30 to 2.05)
Mycophenolate ever used	1.38 (0.58 to 3.29)	-	1.74 (0.70 to 4.34)
Antimalarials ever used	1.47 (0.80 to 2.69)	-	1.39 (0.69 to 2.78)
Glucocorticosteroids (GC) ever	1.39 (0.75 to 2.56)	-	1.03 (0.40 to 2.64)
Cumulative GC^a ^>3.5 g	1.27 (0.75 to 2.17)	-	1.59 (0.69 to 3.67)
Disease activity (second tertile)^b^	1.00 (0.55 to 1.82)	-	1.10(0.56 to 2.14)
Disease activity (third tertile)	0.43 (0.22 to 0.84)	0.49 (0.27 to 0.90)	0.52 (0.24 to 1.12)
Outside North America	1.04 (0.53 to 2.05)	-	1.06 (0.45 to 2.50)
Male	2.47 (1.24 to 4.92)	-	2.21 (1.05 to 4.66)
Age	1.05 (1.03 to 1.07)	1.04 (1.03 to 1.06)	1.05 (1.03 to 1.07)
White race/ethnicity	0.97 (0.55 to 1.71)	-	0.86 (0.46 to 1.59)
Calendar year	1.01 (0.98 to 1.05)	-	0.98 (0.94 to 1.02)
Sjögren's syndrome	1.29 (0.66 to 2.54)	-	1.21 (0.53 to 2.78)

^a^Systemic glucocorticosteroids, considered in prednisone equivalent doses. ^b^Mean adjusted SLE Disease Activity-2K (SLEDAI-2K).

## Conclusion

We did not definitively demonstrate an increased risk for any medications, despite a trend to greater cyclophosphamide use in the lymphoma cases. If anything, we noted a protective effect for very high SLE disease activity. Further work will focus on genetic profiles that might interact with medication exposures to influence lymphoma risk in SLE.

## References

[B1] Bernatsky S Ramsey-Goldman R Labrecque J Joseph L Petri M Zoma A Manzi S Urowitz M Gladman D Fortin PR Ginzler E Yelin E Bae SC Wallace D Edworthy S Barr S Jacobsen S Gordon C Dooley MA Peschken C Hanly J Alarcón G Nived O Ruiz-Irastorza G Isenberg D Rahman A Witte T Aranow C Steinsson K Sturfelt G Cancer risk in systemic lupus: an updated international multi-centre cohort study [brief report] 2012 Submitted 10.1016/j.jaut.2012.12.009 PMC3646904 23410586

